# Equilibrium data for the cross-metathesis of methyl oleate with cinnamaldehyde

**DOI:** 10.1016/j.dib.2018.07.064

**Published:** 2018-07-31

**Authors:** Pablo D. Nieres, Andrés F. Trasarti, Carlos R. Apesteguía

**Affiliations:** Catalysis Science and Engineering Research Group (GICIC), INCAPE, UNL-CONICET, Predio CCT Conicet, Paraje El Pozo, 3000 Santa Fe, Argentina

**Keywords:** Equilibrium data, Cross-metathesis, Methyl oleate, Cinnamaldehyde, Fine chemistry

## Abstract

Here we present equilibrium data for the cross-metathesis of methyl oleate (MO) with cinnamaldehyde (CA) obtained experimentally from liquid-phase catalytic tests conducted at 323 K. The reaction was carried out in batch reactors, using different reactant molar ratios and the 2nd generation Ru Hoveyda-Grubbs complex as catalyst. Reaction mixtures at the equilibrium were analyzed by gas chromatography. Equilibrium constants were determined by assuming unitary activity coefficients for a cinnamaldehyde/methyl oleate equimolar ratio, and the validity of that approximation was evaluated by calculating the equilibrium conversions for different reactant molar ratios.

**Specifications Table**

**Table:** 

Subject area	*Chemical Engineering*
More specific subject area	*Organometallic catalysis, Functionalized Olefin Cross Metathesis*
Type of data	*Figure, Table*
How data was acquired	*Gas Chromatograph, Agilent 6850 Chromatograph (FID). Schlenck-type glass reactor for catalytic tests.*
Data format	*Analyzed*
Experimental factors	*The equilibrium in the cross-metathesis of methyl oleate with cinnamaldehyde was achieved in a Schlenck-type reacter under inert atmosphere at 323 K and different cinnamaldehyde/methyl oleate initial molar ratios.*
Experimental features	*Catalytic tests in batch reactor, monitored by gas chromatography*
Data source location	*INCAPE (Instituto de Investigaciones en Catálisis y Petroquímica), Santa Fe, Argentina*
	https://goo.gl/maps/K8UENtWYqUk
Data accessibility	*Data are accessible with the article*
Related research article	P. Nieres, A.F. Trasarti, C.R. Apesteguía, Valorisation of plant oil derivatives via metathesis reactions: study of the cross-metathesis of methyl oleate with cinnamaldehyde, Mol. Catal. (2018) *in press*

**Value of the data**

• The cross metathesis of methyl oleate with cinnamaldehyde is an attractive route for the synthesis of various useful intermediates and compounds for fine chemistry and polymer industry [1].
• Equilibrium data for MO/cinnamaldehyde reaction (unpublished to date) are relevant to improve the yield in cross-metathesis products, in competition with the MO self-metathesis reaction.
• Theoretical and experimental equilibrium conversions presented here allow other researchers to extend analyses for cross metathesis of unsaturated fatty acid methyl esters with functionalized olefins.

## 1 Data

**Fig. 1:**
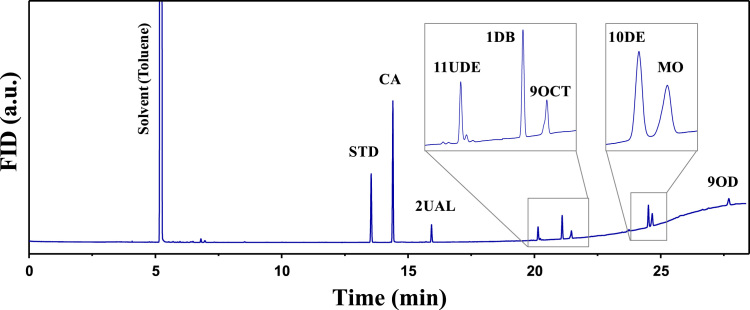
Typical gas chromatogram obtained for the product analysis of MO/cinnamaldehyde metathesis reaction: n-dodecane (standard). CA: cinnamaldehyde, 2UAL: 2-undecenal, 11UDE: methyl 11-oxo-9-undecenoate, 1DB: 1-decenylbenzene, 9OCT: 9-octadecene, 10DE: methyl 10-fenyl-9-decenoate, MO: methyl oleate, 9OD: methyl 9-octadecene-1,18-dioate.

Table 1 lists the retention times and flame ionization detector (FID) response factors relative to n-dodecane (internal standard) for all the reactants and products detected during the catalytic tests. Fig. 1 shows a typical gas chromatogram identifying the reactants methyl oleate (MO) and cinnamaldehyde (CA), and the products 2-undecenal (2UAL), methyl 11-oxo-9-undecenoate (11UDE), 1-decenylbenzene (1DB), 9-octadecene (9OCT), methyl 10-phenyl-9-decenoate (10DE), dimethyl 9-octadecen-1,18-dioate (9OD).

**Table 1:** Retention times and FID response factors for reactants and products.

Substance	Retention time (min)	Response factor^a^
n-dodecane (STD)	13.55	1
cinnamaldehyde (CA)	14.39	0.62
2-undecenal (2UAL)	15.92	0.83
methyl 11-oxo-9-undecenoate (11UDE)	20.14	1.13
1-decenylbenzene (1DB)	21.10	0.80
9-octadecene (9OCT)	21.46	1.38
methyl 10-phenyl-9-decenoate (10DE)	24.51	1.11
methyl oleate (MO)	24.66	1.28
dimethyl 9-octadecen-1,18-dioate (9OD)	27.69	1.20

a Relative to n-dodecane (internal standard)

Table 2 shows the reactions involved in the MO/CA cross-metathesis, while the expressions of the corresponding equilibrium constants $K_{i}^{\mathrm{Eq}}$ are given in Table 3. The experimental values obtained at 323 K for each compound at equilibrium for different initial reactant molar ratios ($R_{\mathrm{CA}/\mathrm{MO}}$) are collected in Table 4. The values of reaction equilibrium constants at 323 K and $R_{\mathrm{CA}/\mathrm{OM}}$=1 were calculated using the experimental data of Table 4 and are presented in Table 5. Finally, we used the $K_{i}^{\mathrm{Eq}}$ values of Table 5 to calculate the theoretical MO equilibrium conversions ($X_{\mathrm{OM}}^{\mathrm{eq}}$)^T^ for $R_{\mathrm{CA}/\mathrm{OM}}$ ratios between 2 and 20 by employing the equilibrium equation system presented in Table 6. The obtained ($X_{\mathrm{OM}}^{\mathrm{eq}}$)^T^ values are compared in Table 7 with the experimental MO equilibrium conversions ($X_{\mathrm{OM}}^{\mathrm{eq}}$)^E^ calculated from data of Table 4.

**Table 2:** Reactions involved in the cross-metathesis of methyl oleate with cinnamaldehyde.

Entry	Equation	Reaction
1	$\mathrm{MO}\overset{K_{1}^{\mathrm{Eq}}}{\Leftrightarrow }\frac{1}{2}9\mathrm{OCT}+\frac{1}{2}9\mathrm{OD}$	MO self-metathesis
2	$\mathrm{MO}+\mathrm{CA}\overset{K_{2}^{\mathrm{Eq}}}{\Leftrightarrow }\frac{1}{2}2\mathrm{UAL}+\frac{1}{2}11\mathrm{UDE}+\frac{1}{2}1\mathrm{DB}+\frac{1}{2}10\mathrm{DE}$	MO/CA cross-metathesis
3	$9\mathrm{OCT}+\mathrm{CA}\overset{K_{3}^{\mathrm{Eq}}}{\Leftrightarrow }2\mathrm{UAL}+1\mathrm{DB}$	9OCT/CA cross-metathesis
4	$9\mathrm{OD}+\mathrm{CA}\overset{K_{4}^{\mathrm{Eq}}}{\Leftrightarrow }11\mathrm{UDE}+10\mathrm{DE}$	9OD/CA cross-metathesis

**Table 3:** Equilibrium constants corresponding to reactions presented in Table 2.

Name	Reaction	$K^{\mathrm{Eq}}$ equation^a^
$K_{1}^{\mathrm{Eq}}$	MO self-metathesis	$K_{1}^{\mathrm{Eq}}=\frac{{[n_{9\mathrm{OCT}}^{\mathrm{eq}}]}^{1/2}.{[n_{9\mathrm{OD}}^{\mathrm{eq}}]}^{1/2}}{[n_{\mathrm{MO}}^{\mathrm{eq}}]}$
$K_{2}^{\mathrm{Eq}}$	MO/CA cross-metathesis	$K_{2}^{\mathrm{Eq}}=\frac{{[n_{2\mathrm{UAL}}^{\mathrm{eq}}]}^{1/2}.{[n_{11\mathrm{UDE}}^{\mathrm{eq}}]}^{1/2}.{[n_{1\mathrm{DB}}^{\mathrm{eq}}]}^{1/2}.{[n_{10\mathrm{DE}}^{\mathrm{eq}}]}^{1/2}}{\left[n_{\mathrm{MO}}^{\mathrm{eq}}\right].[n_{\mathrm{CA}}^{\mathrm{eq}}]}$
$K_{3}^{\mathrm{Eq}}$	9OCT/CA cross metathesis	$K_{3}^{\mathrm{Eq}}=\frac{\left[n_{2\mathrm{UAL}}^{\mathrm{eq}}\right].[n_{1\mathrm{DB}}^{\mathrm{eq}}]}{\left[n_{9\mathrm{OCT}}^{\mathrm{eq}}\right].[n_{\mathrm{CA}}^{\mathrm{eq}}]}$
$K_{4}^{\mathrm{Eq}}$	9OD/CA cross-metathesis	$K_{4}^{\mathrm{Eq}}=\frac{\left[n_{11\mathrm{UDE}}^{\mathrm{eq}}\right].[n_{10\mathrm{DE}}^{\mathrm{eq}}]}{\left[n_{9\mathrm{OD}}^{\mathrm{eq}}\right].[n_{\mathrm{CA}}^{\mathrm{eq}}]}$

a $n_{i}^{\mathrm{eq}}$: moles of compound i at equilibrium

**Table 4:** Experimental values obtained at 323 K for the number moles of reactants and products at equilibrium.

Component	$R_{\mathrm{CA}/\mathrm{OM}}$ (initial CA/MO molar ratio)							
	*1*	*2*	*3*	*4*	*5*	*7*	*9*	*10*
*n-dodecane*^a^	14.5	14.5	14.5	14.5	14.5	14.5	14.5	14.5
*CA*	6.40	17.9	36.3	53.2	67.2	92.4	118.0	125.4
*2UAL*	3.97	6.11	7.11	8.28	7.76	7.90	7.99	7.38
*11UDE*	3.56	5.97	6.00	8.25	7.82	7.79	7.62	6.93
*1DB*	3.96	6.06	6.10	7.99	7.25	6.73	6.34	5.95
*9OCT*	1.56	1.00	0.47	0.46	0.14	0.14	0.16	0.13
*10DE*	3.56	6.06	6.43	7.87	7.35	6.35	6.15	5.65
*MO*	3.10	2.15	1.42	1.22	0.63	0.32	0.31	0.25
*9OD*	1.76	1.37	1.09	0.92	0.72	0.16	0.19	0.15
*BC*^b^	100	98	97	97	99	102	100	97

$C_{\mathrm{MO}}^{0}$= 0.00725 mol/L, W_catalyst_= 2.24 mg, catalyst/MO ratio = 2.38% molar, solvent: toluene (10 mL)

a Internal standard

b Carbon balance, calculated at equilibrium

**Table 5:** Equilibrium constants at 323 K calculated using the values of Table 4 at $R_{\mathrm{CA}/\mathrm{OM}}$=1.

Reaction	$K^{\mathrm{Eq}}$ value
$\mathrm{OM}\overset{K_{1}^{\mathrm{Eq}}}{\Leftrightarrow }\frac{1}{2}9\mathrm{OCT}+\frac{1}{2}9\mathrm{OD}$	$K_{1}^{\mathrm{Eq}}$ = 0.53
$\mathrm{OM}+\mathrm{CA}\overset{K_{2}^{\mathrm{Eq}}}{\Leftrightarrow }\frac{1}{2}2\mathrm{UAL}+\frac{1}{2}11\mathrm{UDE}+\frac{1}{2}1\mathrm{DB}+\frac{1}{2}10\mathrm{DE}$	$K_{2}^{\mathrm{Eq}}$ = 0.71
$9\mathrm{OCT}+\mathrm{CA}\overset{K_{3}^{\mathrm{Eq}}}{\Leftrightarrow }2\mathrm{UAL}+1\mathrm{DB}$	$K_{3}^{\mathrm{Eq}}$ = 1.58
$9\mathrm{OD}+\mathrm{CA}\overset{K_{4}^{\mathrm{Eq}}}{\Leftrightarrow }11\mathrm{UDE}+10\mathrm{DE}$	$K_{4}^{\mathrm{Eq}}$ = 1.13

**Table 6:** Equilibrium equations used to validate experimental $K^{\mathrm{Eq}}$ values.

*Mass Balance*	$K^{\mathrm{Eq}}$*equations*
$n_{\mathrm{OM}}^{\mathrm{eq}}=n_{\mathrm{MO}}^{0}-\varepsilon_{1}-\varepsilon_{2}$	$K_{1}^{\mathrm{Eq}}=\frac{{\left(\varepsilon_{1}/2-\varepsilon_{3}\right)}^{1/2}{\left(\varepsilon_{1}/2-\varepsilon_{4}\right)}^{1/2}}{n_{\mathrm{MO}}^{0}-\varepsilon_{1}-\varepsilon_{2}}$
$n_{\mathrm{CA}}^{\mathrm{eq}}=n_{\mathrm{CA}}^{0}-\varepsilon_{2}-\varepsilon_{3}-\varepsilon_{4}-\varepsilon_{4}$	
$n_{9\mathrm{OD}}^{\mathrm{eq}}=\varepsilon_{1}/2-\varepsilon_{4}$	$K_{3}^{\mathrm{Eq}}=\frac{{\left(\varepsilon_{2}/2+\varepsilon_{3}\right)}^{2}}{\left(\varepsilon_{1}/2-\varepsilon_{3}\right)(R_{\mathrm{CA}/\mathrm{OM}}.n_{\mathrm{OM}}^{0}-\varepsilon_{2}-\varepsilon_{3}-\varepsilon_{4}-\varepsilon_{5})}$
$n_{2\mathrm{UAL}}^{\mathrm{eq}}=\varepsilon_{2}/2+\varepsilon_{3}$	
$n_{1\mathrm{DB}}^{\mathrm{eq}}=\varepsilon_{2}/2+\varepsilon_{3}$	$K_{4}^{\mathrm{Eq}}=\frac{{\left(\varepsilon_{2}/2\varepsilon_{2}+\varepsilon_{4}\right)}^{2}}{\left(\varepsilon_{1}/2-\varepsilon_{4}\right)(R_{\mathrm{CA}/\mathrm{MO}}.n_{\mathrm{OM}}^{0}-\varepsilon_{2}-\varepsilon_{3}-\varepsilon_{4}-\varepsilon_{5})}$
$n_{11\mathrm{UDE}}^{\mathrm{eq}}=\varepsilon_{2}/2+\varepsilon_{4}$	$X_{\mathrm{MO}}^{\mathrm{eq}}=\frac{\varepsilon_{1}+\varepsilon_{2}}{n_{\mathrm{MO}}^{0}}$
$n_{10\mathrm{DE}}^{\mathrm{eq}}=\varepsilon_{2}/2+\varepsilon_{4}$	

$\varepsilon_{i}$*: reaction extent for reaction*i*(see*Table 2*)*

$X_{\mathrm{OM}}^{\mathrm{eq}}$*: MO conversion at equilibrium*

**Table 7:** Theoretical and experimental MO equilibrium conversions for the MO/CA cross-metathesis reaction.

Reactant ratio (molar) $R_{\mathrm{CA}/\mathrm{OM}}$	Equilibrium MO conversion (%)	
	Theoretical ${(X}_{\mathrm{MO}}^{\mathrm{eq}}$)^T^	Experimental ($X_{\mathrm{MO}}^{\mathrm{eq}}$)^E^
1	79	79
2	86	87
3	93	91
4	94	94
5	95	96
7	96	97
9	96	99
10	96	100

$C_{\mathrm{MO}}^{0}$= 0.00725 mol/L, W_catalyst_= 2.24 mg, catalyst/MO ratio = 2.38% molar, solvent: toluene (10 mL) T = 323 K

## 2 Experimental design, materials, and methods

### 2.1 Materials

Methyl oleate (Aldrich, 99%), cinnamaldehyde (Aldrich, 99%), n-dodecane (Aldrich, >99%), toluene (Sigma-Aldrich, 99.8%), HG2 complex (Aldrich, 97%), benzophenone (Aldrich, 99%), metallic Na (Tetrahedron, 99%), methanol, absolute (Merck).

### 2.2 Catalytic tests

MO/CA cross-metathesis catalytic tests were performed under argon in a Schlenk flask at atmospheric pressure and 323 K. The solvent (toluene) was previously dehydrated under reflux with sodium/benzophenone in dry argon atmosphere. The reactor was loaded with 10 ml of solvent, the internal standard and suitable amounts of reactants (methyl oleate and cinnamaldehyde). After heating the reaction mixture to the reaction temperature (thermostatic bath) the catalyst (2nd generation Hoveyda-Grubbs complex, 2.24 mg) was added to start the reaction. Samples taken from the reactor after reaching the equilibrium were collected into an ice-cooled vial containing methanol, to ensure that the reaction was quenched. The thermodynamic equilibrium was verified by introducing into the reactor an additional amount (1.2 mg) of fresh catalyst at the end of the 7-h catalytic run and checking that MO conversion was not modified by the addition of catalyst.

### 2.3 Chromatographic analysis

Samples from catalytic test were analyzed by gas chromatography using an Agilent 6850 equipped with a Flame Ionization Detector (FID). Chromatographic conditions are summarized in Table 8.

**Table 8:** Chromatographic conditions used in the analysis of samples from catalytic tests.

Inyector (split/splitless)	Detector (FID)	Oven
Mode: split	Temperature: 583 K	Column: HP-1
Temperature: 573 K	H_2_ flow: 60 cc/min	Length: 50 m
Split ratio: 40	Air flow: 450 cc/min	Diameter: 0.32 mm
Purge flow: 60 cc/min	Make up gas: N_2_	Film thickness: 1.50 μm
Purge time: 0.75 min	Make up gas flow: 50 cc/min	Mode: temperature ramp
		Initial T: 373 K
		Initial time: 0 min
		Temperature rate: 10 K/min
		Final T: 573 K
		Final time: 15 min
		Carrier gas: N_2_
		Carrier gas flow: 1.61 cc/min

## 3 Data analysis

Response factors ($f_{i}$) relative to the internal standard (n-dodecane) were calculated from chromatographic analysis according to Eq. (1):

(1) $$f_{i}=\frac{n_{i}\;.\;A_{\mathrm{std}}}{A_{i}\;.\;n_{\mathrm{std}}}$$

where $n_{i}$ and $n_{\mathrm{std}}$ are the moles of component i and the standard, respectively, while $A_{\mathrm{std}}$ and $A_{i}$ are the integrated chromatographic areas of the standard and the i component, respectively. When chromatographic standards were not commercially available, $f_{i}$ was determined from the carbon balance using samples with different compositions.

The number of moles of component *i* at equilibrium was calculated from Eq. (2):

(2) $$n_{i}^{\mathrm{Eq}}=f_{i}\frac{A_{i}^{\mathrm{Eq}}}{A_{\mathrm{std}}}n_{\mathrm{std}}$$

where $n_{i}^{\mathrm{Eq}}$ and $A_{i}^{\mathrm{Eq}}$ are the moles and the integrated chromatographic area of component *i* in the reaction mixture at equilibrium, respectively.

Carbon balance was calculated according to Eq. (3):

(3) $$\mathrm{BC}=\frac{\alpha_{\mathrm{OM}}*n_{\mathrm{MO}}+\gamma_{\mathrm{CA}}*n_{\mathrm{CA}}+\sum_{i}\omega_{i}*n_{i}}{\alpha_{\mathrm{MO}}*n_{\mathrm{MO}}^{0}+\gamma_{\mathrm{CA}}*n_{\mathrm{CA}}^{0}}*100$$

where $\alpha_{\mathrm{OM}}$ are the number of C atoms in MO molecule, $\gamma_{\mathrm{CA}}$ the number of C atoms in CA molecule, and $\omega_{i}$ the number of C atoms in the *i*-product molecule; $n_{\mathrm{OM}}^{0}$ and $n_{\mathrm{OM}}$ are moles of MO at time *0* and at equilibrium, respectively, while $n_{\mathrm{CA}}^{0}$ and $n_{\mathrm{CA}}$ indicate the corresponding moles of CA.
